# Supplementary material for: The genome sequence of the Brown Moss-moth, Bryotropha terrella (Denis & Schiffermüller), 1775
Source: Wellcome Open Res. 2025 Jun 4;10:310. [Version 1] doi: 10.12688/wellcomeopenres.24324.1 (PMC12402767; doi:10.12688/wellcomeopenres.24324.1)
Supplement: Peer review history

# Reviewer report: Reviewer response for version 1

**DOI:** 10.21956/wellcomeopenres.26825.r127593

**Published:** 2025-09-01

**Authors:** Annabel Whibley

**Affiliations:** 1 Grapevine Improvement, Bragato Research Institute, Lincoln, New Zealand

In this Data Note, Hutchinson, Crowley, Broad and colleagues report the genome assembly of the Brown Moss-moth, *Bryotropha terrella. * No genome annotation is presented. **I will note, really as a counterpoint to other data notes I have reviewed or read, that this makes little comment on the natural history of the species, including only the Darwin Tree of Life goals as justification for the work. At the very least I would like some steer as to whether this is due to lack of knowledge or lack of interest.

 The quality of the assembly is excellent, with very few contigs and >99.6% of the assembly scaffolded into chromosomes, and impressive QV and completeness metrics.  Chromosome 1 is markedly larger than the Z and other autosomal scaffolds, which strikes me as unusual, and I would be interested to view this alongside other related species: it shows some HiC interactions with the W chromosome and is perhaps enriched in repeat elements?

 The methods and reporting follow standard Darwin Tree of Life templates. The links to publicly available  datasets are functional, and report of methods and metadata is comprehensive and well-structured.

Are sufficient details of methods and materials provided to allow replication by others?

Yes

Is the rationale for creating the dataset(s) clearly described?

Yes

Are the datasets clearly presented in a useable and accessible format?

Yes

Are the protocols appropriate and is the work technically sound?

Yes

Reviewer Expertise:

Genomics, Bioinformatics, Evolution

I confirm that I have read this submission and believe that I have an appropriate level of expertise to confirm that it is of an acceptable scientific standard.

# Reviewer report: Reviewer response for version 1

**DOI:** 10.21956/wellcomeopenres.26825.r127587

**Published:** 2025-09-01

**Authors:** Panagiotis Ioannidis

**Affiliations:** 1 Foundation for Research & Technology - Hellas, Crete, Greece

This manuscript describes the sequencing and assembly of the genome of the lepidopteran Bryotropha terrela. The methodology used is the standard methodology used in all DToL samples, which is producing high quality genome assemblies. Such assemblies are suitable for any type of comparative analyses that aim to elucidate the ecology and evolution of this organism.

 More specifically, assembly contiguity is very good since virtually all contigs were assigned to a chromosome. Additionally, BUSCO scores suggest that the content of the contigs is excellent.

 My only comment has to do with the blobplot. Looking at it (figure 3) I noticed that there are quite a few small contigs either of low coverage, or of high GC content. All of them are annotated as "no-hit". Also, there is a couple of large contigs that are annotated as "no-hit". Could the authors comment on the origin of these contigs? Could they be symbiont related?

Are sufficient details of methods and materials provided to allow replication by others?

Yes

Is the rationale for creating the dataset(s) clearly described?

Yes

Are the datasets clearly presented in a useable and accessible format?

Yes

Are the protocols appropriate and is the work technically sound?

Yes

Reviewer Expertise:

insect genomics, bioinformatics

I confirm that I have read this submission and believe that I have an appropriate level of expertise to confirm that it is of an acceptable scientific standard.

# Reviewer report: Reviewer response for version 1

**DOI:** 10.21956/wellcomeopenres.26825.r125101

**Published:** 2025-06-25

**Authors:** Sivasankaran Kuppusamy

**Affiliations:** 1 Loyola College, Chennai, Tamil Nadu, India

Authors have done the chromosome-level genome sequence of *Bryotropha terrella* ([Denis & Schiffermüller], 1775). They have observed 31 chromosomal pseudomolecules in the assembly.

**Minor comments on the Manuscript**

 Authors haven’t given the proper taxonomic parenthesis of the describer of the species in the title.  The title can be modified as “The genome sequence of the Brown Moss-moth, *Bryotropha terrella *([Denis & Schiffermüller], 1775)”

 Authors can include brief bionomics of the sequenced species in the background of the article.   

 Authors have used the full form of Genus name *Bryotropha *throughout the text. The genus name can be used in full form first time and the can be a short form like *B. terrella.*

 Authors have produced 100.43 Gb comprehensive sequences through the Hi-C illumina platform and PacBio. After the assembly they received 756.35 megabases. But authors haven’t observed the protein-coding genes, non-coding genes and gene transcripts. Any reason?

 The research article was well prepared and the manuscript meets the necessary scientific standard and is suitable for indexing

Are sufficient details of methods and materials provided to allow replication by others?

Yes

Is the rationale for creating the dataset(s) clearly described?

Yes

Are the datasets clearly presented in a useable and accessible format?

Yes

Are the protocols appropriate and is the work technically sound?

Yes

Reviewer Expertise:

Phylogenetic analysis of Noctuoidea moths using mitogenome sequence

I confirm that I have read this submission and believe that I have an appropriate level of expertise to confirm that it is of an acceptable scientific standard.
